# Coping Humor of Entrepreneurs: Interaction Between Social Culture and Entrepreneurial Experience

**DOI:** 10.3389/fpsyg.2018.01449

**Published:** 2018-08-29

**Authors:** Song Lin, Jing Li, Rui Han

**Affiliations:** Department of Strategy, Central University of Finance and Economics, Beijing, China

**Keywords:** coping humor, social culture, entrepreneurial failure, business performance, entrepreneurial experience

## Abstract

It is feasible to deal with the high pressures in entrepreneurship using humor. This paper studies the effect of interaction between entrepreneurs’ perception of social culture and entrepreneurial experience (including experience with entrepreneurial failure and current company performance) on coping humor of entrepreneurs with a sample of 171 entrepreneurs from Bohai Rim in China. Regression analysis revealed that entrepreneurs would be more likely to adopt coping humor when they perceived supportive social culture to entrepreneurship and had experienced entrepreneurial failure or when they perceived supportive social culture to entrepreneurship and had good current business. This study contributes to the literature of the theory of humor, the culture of entrepreneurship, and entrepreneurial failure.

## Introduction

Humor is usually considered multifunctional. It helps individuals mitigate tension and anxiety by managing emotions, thus promoting their physical and mental health (Lefcourt, 2001; Martin, 2001; Marziali et al., 2008). In recent years, increased attention has been paid to humor, which is considered an important coping strategy for dealing with stress and adversity (Geisler et al., 2009; Sliter et al., 2014; Morse et al., 2018), and such humor targeted at coping with stress is called coping humor (Martin and Lefcourt, 1983; Martin, 2001). Coping humor helps individuals to reassess stressful events in a positive way by seeking the bright side (Martin et al., 1993), which can solve the problem by changing the stress itself (Lefcourt et al., 1997). Therefore, coping humor is commonly used to deal with pressure (Martin, 2007, 2016; Merz et al., 2009; Demjén, 2016; Morse et al., 2018).

Entrepreneurs confront high pressure (Xie et al., 2008; Manzano-García and Ayala Calvo, 2013). Compared with established enterprises, startups – as new entrants into the marketplace – usually lack market power and resources; they are often weak in legitimacy, information symmetry, and performance record (Bhidé, 2000; Shane and Cable, 2002; Bruton et al., 2010), which add to the uncertainties of the business environment (Schendel, 2007; McKelvie et al., 2011). Some entrepreneurs have already tried to use humor as a way to address pressure or adversity. Ma Yun, a successful entrepreneur and founder of Alibaba – the largest e-commerce platform in China – is famous for his skillful self-mockery and humorous imagery (Liu, 2014). By taking advantage of self-mockery and humor, Ma has managed to cope with many stressful situations in his business and has won extensive public support (Kim et al., 2016). Nevertheless, entrepreneurs are considered grave and unamiable in most cases (Gupta et al., 2005, 2009; Akhtar et al., 2013). What kinds of factors may affect entrepreneurs’ decisions to cope with stress through humor? Few studies have explored this issue in entrepreneurship research, but this paper will try to fill the gap.

In previous studies, some scholars have pointed out that entrepreneurs’ stress-coping strategies are affected by factors such as social culture (Liu and Almor, 2016), perceived stress of individuals (Anderson, 1975), personal traits (Connor-Smith and Flachsbart, 2007), and type of stress (Schonfeld and Mazzola, 2015). In studies on humor, cultural and individual factors are also commonly mentioned as influential components of an individual’s use of coping humor (Martin, 2007; Yue et al., 2016). Some studies suggest that people living in specific cultures hold different attitudes toward humor (Yue et al., 2016), which may lead to encouragement or discouragement for using humor as a way to deal with pressure (Wu and Chan, 2013). In addition, various subjective experiences may also affect the use of coping humor. Chapple and Ziebland (2004) showed that patients with incurable diseases tend to be more sensitive and negative to others’ humorous take on their situation on them than other patients, and they were less active in resolving stress with humor as well. These studies provide research clues for this paper.

This paper studies the effects of social culture sensed by entrepreneurs and their entrepreneurial experience on their use of humor to cope. The study uses entrepreneurs from Bohai Rim in China as the sample set to check the research hypotheses. The study may make some contributions listed in the following aspects: first, it studies situations in which entrepreneurs would find it helpful to adopt coping humor. The effect of culture and experience on coping humor shown in the study reveals the mechanism by which individuals adopt humor when confronting stress. Because this has not been fully researched in previous studies on humor, the present study may contribute to the insight of prior theories in this field. Second, the effect of social culture sensed by entrepreneurs in their use of coping humor actually enriches the role of social culture in the entrepreneurial process, thus contributing to the theoretical study of entrepreneurship culture. Third, the entrepreneurs’ experience discussed in this paper includes entrepreneurial failure. Previous studies on the effect of failure focused mainly on learning from failure, whereas this paper explores the relation between experience of failure and the use of humor, which connects the experience of failure with stress coping mechanisms used by entrepreneurs, thus contributing to the literature on entrepreneurial failure.

## Theories and Hypotheses

### Coping Humor and Coping Theory

Coping humor is defined as the propensity to use humor as a method for individuals to cope with stress or adversity in life (Martin and Lefcourt, 1983; Martin, 2007). Compared with other positive stress-coping strategies, like rational actions or self-adjustment, coping humor may help ease stress by improving individual mental happiness and personal charm and creating a friendly atmosphere (Martin, 2007). Because humor as a coping mechanism involves making an effort to keep a humorous perspective in the face of adversity (Martin, 1996, 2007; Marziali et al., 2008; Merz et al., 2009), coping theory can be used to explain the connection between social culture, entrepreneurial experience, and entrepreneurs’ coping humor.

Coping theory considers coping as “constantly changing cognitive and behavioral efforts to manage specific external and/or internal demands that are appraised as taxing or exceeding the resources of the person” (Lazarus and Folkman, 1984, p. 141). The theory stresses that both the selection and effect of coping strategies depend on the interaction between individuals’ perceived stress and their sense of control over stress (Lazarus and Launier, 1978; Lazarus and Folkman, 1984). If individuals’ perceived stress levels are high and uncontrollable because of perceived deficiencies in their capabilities and resources, they will feel hopeless or angry, thus concentrating too much on negative coping mechanisms like emotions or defense (Anderson, 1977; Lazarus and Launier, 1978; Lazarus and Folkman, 1984). However, if their perceived stress is moderate and controllable with their capabilities and resources, they will consider the stress a challenge instead of a threat (Lazarus and Folkman, 1984), which would reduce their anxiety and encourage them to make active preparations and to use more proactive coping strategies to deal with stress (Lazarus and Launier, 1978).

In conclusion, both perceived stress and control over said stress codetermine individual selection of coping strategies and subsequent effects. Based on this theory, the following exposition explores the effect of the interaction between the social culture perceived by entrepreneurs (affecting the extent of perceived stress) and entrepreneurial experience, including entrepreneurial experience of failure and current business performance (affecting entrepreneurs’ sense of control over stress) on entrepreneurs’ coping humor.

### Social Culture and Coping Humor

Regarding the degree of perceived stress, this paper is mainly concerned with the social culture that reflects the degree to which society supports and understands entrepreneurship, for this is closely related to the implementation of entrepreneurial activities. Social culture, as a type of institutional environment, is usually composed of values on what is considered proper and norms on how to behave in accordance with these values (Scott, 2007). The social culture in support of entrepreneurship reflects members’ appreciation of entrepreneurship in a certain country or region (Busenitz et al., 2000). If entrepreneurs sense that the views of people around them are unfavorable toward entrepreneurship, they will view it as difficult to win physical and mental support from the social environment for their entrepreneurship and may anticipate that they will face serious repercussions in the face of failure, which creates an even greater pressure (Yamakawa et al., 2015). Conversely, when entrepreneurs find the social culture favorable to entrepreneurship, they will generally feel less stress when carrying out entrepreneurial activities. Moreover, a moderate stress level (as opposed to a high stress level) would encourage people to be more proactive to cope with the stressful situation (Anderson, 1975). This means that when social culture is favorable to entrepreneurship, entrepreneurs may be motivated to apply coping humor, which – according to coping theory – requires the interaction between social culture and a sense of control over stress to generate a desirable effect.

### The Interaction Between Social Culture and Entrepreneurial Failure Experience on Coping Humor

In a society where social culture is favorable to entrepreneurship, entrepreneurs who have previously experienced entrepreneurial failures are more likely to use coping humor to address stress. This is because, on the one hand, if social culture as perceived by entrepreneurs is favorable to entrepreneurship, they will experience goodwill in such an environment and bear less mental stress on the occasion of entrepreneurial failure. In such a case, they have no need to worry about tremendous pressure for making errors in entrepreneurial actions; thus, they can feel encouraged to take more proactive strategies with an open and positive mind regarding coping stress. On the other hand, those failures contribute to a stronger sense of control of entrepreneurs over entrepreneurial stress. Failures offer entrepreneurs chances to reflect on and reevaluate their entrepreneurial capabilities (Cope, 2011). Their subsequent endeavors, after a failure, show their positive assessment of their capabilities, better expectations for future entrepreneurship, and their resilience in the face of adversity like failure (Benight and Bandura, 2003; Manzano-García and Ayala Calvo, 2013). Additionally, entrepreneurs can draw lessons from their failures and gain abundant knowledge and new skills to cope with difficulties and setbacks in the future (Cope, 2011; Yamakawa et al., 2015). The above capabilities and resources help strengthen entrepreneurs’ control over prospective setbacks in their future entrepreneurial endeavors. They enhance the feasibility of the active strategies of entrepreneurs triggered by a social culture favorable to entrepreneurship, prompting entrepreneurs to take more active strategies to cope with stress.

Among numerous positive coping strategies, coping humor helps entrepreneurs deal with stressful events and adds to entrepreneurs’ mirth and personal charisma (Lefcourt and Martin, 1986; Martin et al., 1993; Kuiper et al., 1995; Martin, 2016). This explains why coping humor can be adopted as a better or more efficient approach to entrepreneurship stresses in the interaction between social culture and sense of control over stress. Accordingly, we propose the following hypothesis:

**Hypothesis 1**: If entrepreneurs perceive social culture to be favorable to entrepreneurship and they have experienced entrepreneurial failure, they prefer coping with stress by using humor.

### The Interaction Between Social Culture and Business Performance on Coping Humor

In a society where social culture is favorable to entrepreneurship, entrepreneurs whose business is currently in good standing are more likely to use coping humor to address stress. As previously mentioned, entrepreneurs perceive relatively low stress in such a society and are better motivated to take active strategies to cope with stress. Additionally, business performance also represents the entrepreneurs’ capability to control entrepreneurial stress. Entrepreneurs with good business performance may have better control over stressful events, as good performance may indicate more material resources and access to more qualified employees (Singh and Mitchell, 2005; Pe’er et al., 2016), or it may represent better capability in managing business activities, which promotes the entrepreneurs’ perception of self-efficacy (Bandura, 1997; Yamakawa et al., 2015) and consequently strengthens their sense of control over stressful events (Markman et al., 2002). As a result, entrepreneurs will feel more confident when dealing with stressful events (Markman et al., 2002). Therefore, when performance is good, it will also make it more feasible for entrepreneurs to use active strategies in a social culture favorable to entrepreneurship. They will also adopt more positive strategies to deal with pressure. According to the analysis of Hypothesis 1, we also propose Hypothesis 2.

**Hypothesis 2**: If entrepreneurs perceive social culture to be favorable to entrepreneurship and their current businesses are performing well, they prefer coping with stress by using humor.

## Samples and Scale

### Samples

The data used to verify our hypotheses come from two surveys of business founders located in five cities in the Bohai Rim area (Beijing, Tianjin, Shandong, Hebei, and Liaoning). The Bohai Rim area was selected because it is in Northern China and Northeastern China, where the economy is relatively advanced, and entrepreneurship has developed in a more active way in these areas than in inland areas and Western China (Gao et al., 2005). In addition, this area is sprinkled with new ventures, a condition that offers dedicated support for our entrepreneurship study.

From September to December 2016, we formed an investigation team and conducted the face-to-face interviews with business founders in the area. This type of research enabled investigators to offer accurate explanations of the questions and options in the questionnaire, making its quality evident. This research obtained 989 samples. From September to December 2017, we conducted follow-up investigations into the 638 sample enterprises from the original set that could be reached. Enterprises operating for over 8 years and those with a lack of key variables were screened out, resulting in a sample size of 171 startups. Independent variables were collected from the 2016 data, and dependent variables were collected from the 2017 data.

Ethics approval for this study was obtained from the Academic Committee of the Business School of the Central University of Finance and Economics, China. The participants participated voluntarily. Written informed consent had been obtained from all participants before their participation, and they had been informed that they had the right to withdraw or terminate the interviews at any time. Additionally, the business information and individual privacy of the participants have been kept confidential.

The characteristics of the samples are shown in **Table 1**. Male entrepreneurs represented the larger share of the sample, with a proportion of 61.4%, and the average age of the sample was 39.98 years. With regard to educational background, most entrepreneurs graduated from junior colleges and senior high schools/specialized secondary schools. The former group accounted for the highest percentage at 36.8%, followed by graduates from senior high schools/specialized secondary schools with a percentage of 35.7%. Entrepreneurs with no entrepreneurial failures represented the largest percentage at 67.8%. The present average age of enterprises was 4.59 years. In addition, the distribution of samples showed that Shandong Province had the most startups in the sample, accounting for 31% of all businesses surveyed. It was followed by Tianjin at 26.9%; Liaoning had the smallest proportion at 7.6%.

**Table 1:** Characteristics of samples.

Characteristics of samples	Distribution
**Gender**	
Male	61.4%
Female	38.6%
**Educational background**	
Junior high school graduates	2.9%
Senior high school/specialized secondary school graduates	35.7%
Junior college graduates	36.8%
Bachelor’s degree	23.4%
Master’s degree	0.6%
Doctorate	0.6%
**Experience of entrepreneurial failure**	
Yes	32.2%
No	67.8%
**Business location**	
Beijing	19.9%
Tianjin	26.9%
Hebei	14.6%
Shandong	31.0%
Liaoning	7.6%
Characteristics of samples	Average
Age of entrepreneurs	39.98 years
Corporate duration	4.59 years


### Scale

#### Dependent Variable

##### Coping humor

The study used a single item to evaluate the extent to which the entrepreneur uses humor to cope with pressure: “I usually look for something comical to say when I am in tense situations.” The item comes from the coping humor scale developed by Martin and Lefcourt (1983). The respondents were required to answer how he or she agreed or disagreed with this item on a 5-point Likert-type scale (1 stands for totally disagree, and 5 stands for totally agree). According to existing literature, if the content of a construct being measured is unidimensional and mainly reflects a subjective experience of participants (Robins et al., 2001) or is clear to participants (Wanous et al., 1997), then a single item can be used to measure a construct (Wanous et al., 1997; Robins et al., 2001). Moreover, many previous researchers have used a single item to evaluate the major variable (Nett et al., 2011; Goetz et al., 2014; Goldberg-Looney et al., 2016).

In addition, we believe that measuring with a single item outperforms the general scale developed by Martin and Lefcourt (1983), as existing researchers find that participants have different explanations for some items in such a scale. For instance, “I must admit my life would probably be easier if I had more of a sense of humor” conflicts with other items (Martin, 1996) because it only manifests the expected sense of humor of an individual but cannot actually reflect the extent to which the individual used humor to cope with pressure in the past. Another example is the item that states, “It has been my experience that humor is often a very effective way of coping with problems,” which emphasizes an individual’s experience-based understanding but does not tell if, or to what extent, he or she will use humor when faced with pressure. The single dimension applied in our research exactly reflects the participant’s experience of coping humor under pressure, and it can be clearly explained to the participant. Furthermore, a measure of construct with a single item also has an advantage in large-scale surveys and vertical longitudinal studies, which this paper adopts.

#### Independent Variables

##### Social culture

In this paper, social culture refers to the culture that supports entrepreneurial activities in the region. The authors adopted the normative dimension in Busenitz et al.’s (2000) institutional environment scale for entrepreneurship to evaluate the social culture perceived by the entrepreneur. The dimension includes four items to assess people’s attitudes toward and evaluation of entrepreneurial activities in the region: (a) turning new ideas into businesses is an admired career path in this region; (b) in this region, innovative and creative thinking is viewed as the route to success; (c) entrepreneurs are admired in this region; and (d) people in this region tend to greatly admire those who start their own business. Participants were required to answer whether he or she agreed with these items on a 5-point Likert-type scale, with 1 standing for “highly disagree” and 5 standing for “highly agree.” In the study of Busenitz et al. (2000), the Cronbach’s alpha of such a dimension is 0.810. Our study has a Cronbach’s alpha of 0.695, close to 0.70, meaning that the scale’s level of reliability is acceptable. In addition, the paper also included a confirmatory factor analysis to test the construct’s validity. The results (CMIN = 22.686, DF = 2, GFI = 0.935, CFI = 0.835) prove that the scale has acceptable validity.

##### The experience of entrepreneurial failure

The researchers required participants to answer whether they have experienced a business closure or sale in a previous company (Yes = 1, No = 0). Because there is no single, agreed-upon definition of failure in the existing literature (Ucbasaran et al., 2013) and some definitions cannot be directly measured, we used the above measurement according to previous empirical literature to reflect the experience of entrepreneurial failure (Ucbasaran et al., 2006, 2010).

##### Business performance

This refers to the business performance of the current enterprise, represented by the sales growth rate of their current companies over the past 3 years based on reports from entrepreneurs. The goal of an entrepreneur is usually to focus on growth rather than profit (Wiklund et al., 2003). In addition, sales growth, mainly reflecting the level of acceptance of products or services, is the most fundamental performance indicator of an enterprise’s activities (Murphy et al., 1996; Ensley et al., 2002), and it has been widely used in previous studies (Baum et al., 1998; Ensley et al., 2002; Gilbert et al., 2006).

#### Control Variables

The authors took some demographic characteristics of entrepreneurs as control variables (i.e., age, gender, educational level, and number of siblings), which are commonly seen in articles on personalities or emotions (Toman, 1992; Celso, 2003; Wu and Chan, 2013; Avloniti et al., 2014). Among these, *age* was represented by the real age of the interviewee; *gender* was measured by the dummy variable 0–1 (1 for male, 0 for female); *educational level* was measured by eight categories (1 for primary school graduates, 2 for junior high school graduates, 3 for senior high/specialized secondary school graduates, 4 for junior college graduates, 5 for undergraduates, 6 for master graduates, 7 for doctoral graduates, and 8 for others); *number of siblings* was represented by the real number of brothers and sisters of each interviewee. The authors also took certain corporate characteristics into account, namely, the age of the enterprise, corporate size, and corporate location (Ucbasaran et al., 2010; Kolympiris et al., 2015; Yamakawa et al., 2015). *The age of enterprise* referred to how long the enterprise has operated to date, represented by the number of years that have passed since the enterprise was founded. The age of the enterprise may indicate whether the enterprise is influenced by the liability of newness or outdated disadvantages, as well as the level of corporate legitimacy (Ucbasaran et al., 2010). *Corporate size*, measured by the natural logarithm of its asset size, can reflect the amount of available resources (Carayannopoulos, 2009; Fang et al., 2016). *Corporate location* was the region where its headquarters is based, and it falls into five provinces and municipalities: Beijing, Tianjin, Hebei, Shandong, and Liaoning. It is measured by four dummy variables: Beijing (Yes = 1, No = 0), Tianjin (Yes = 1, No = 0), Hebei (Yes = 1, No = 0), and Shandong (Yes = 1, No = 0). Corporate location reflects diverse cultural norms, infrastructure, and knowledge asset backgrounds in different regions (Cardon et al., 2011; Kolympiris et al., 2015). All these factors may influence the pressure that entrepreneurs perceive and their sense of control over pressure.

## Statistical Results and Analysis

### Descriptive Statistics and Relevant Analysis

We conducted descriptive analysis and correlation analysis for all relevant variables, as shown in **Table 2**. The correlation coefficients between different variables are moderate, with no significantly high ones, which indicates that there is no obvious collinearity between variables, and further regression analysis is possible.

**Table 2:** Means, SD, and correlations.

	Mean	SD	1	2	3	4	5	6	7	8	9	10	11	12	13	14
1 Coping humor	4.370	0.710	1													
2 Social culture	3.678	0.632	0.138	1												
3 Experience of entrepreneurial failure	0.322	0.468	-0.111	-0.026	1											
4 Business performance	14.320	11.502	0.082	0.119	0.040	1										
5 Age	39.980	8.428	-0.001	0.021	0.362**	0.157*	1									
6 Gender	1.390	0.488	0.113	0.143	-0.134	0.049	-0.084	1								
7 Education level	3.850	0.875	0.024	0.055	-0.181*	0.128	-0.439**	-0.055	1							
8 Number of siblings	2.800	0.865	0.005	-0.058	0.173*	0.023	0.074	-0.040	-0.141	1						
9 Age of enterprise	4.591	2.091	0.082	0.028	-0.171*	0.178*	0.139	0.075	-0.057	0.004	1					
10 Corporate size	5.045	1.666	0.015	0.079	0.075	0.590**	0.140	0.000	0.177*	0.127	0.229**	1				
11 Beijing	0.199	0.400	0.051	0.173*	-0.123	0.234**	-0.136	0.056	0.288**	-0.089	-0.036	0.241**	1			
12 Tianjin	0.269	0.445	-0.036	-0.020	-0.022	-0.319**	-0.009	0.061	-0.121	0.002	-0.052	-0.217**	0.028	1		
13 Hebei	0.146	0.354	-0.028	-0.025	0.140	-0.042	0.021	0.012	-0.118	0.019	-0.006	0.012	-0.206**	-0.251**	1	
14 Shandong	0.310	0.464	0.026	-0.039	-0.055	0.048	0.024	-0.194*	0.131	0.081	0.028	0.095	-0.334**	-0.407**	-0.277**	1


*^∗^*p* < 0.05, ^∗∗^*p* < 0.01.*

### Regression Analysis

Based on the theoretical hypotheses constructed in this paper, we ran hierarchical regression, as shown in **Table 3**. Model 1 is used to evaluate the effect of control variables on the dependent variable. In Model 2, three variables – social culture, the experience of entrepreneurial failure, and business performance –were added to test the effect of independent variables on the dependent variable. In Model 3, the interaction terms between social culture and the experience of entrepreneurial failure, as well as between social culture and business performance, were added to test the interactive effects.

**Table 3:** Regressive results of different models.

Variables	Model 1	Model 2	Model 3
**Control variables**			
Age	0.002 (0.008)	0.003 (0.008)	0.003 (0.008)
Gender	0.175 (0.117)	0.131 (0.119)	0.145 (0.117)
Education level	0.017 (0.078)	0.013 (0.078)	-0.018 (0.077)
Number of siblings	0.015 (0.066)	0.031 (0.067)	0.021 (0.066)
Age of the enterprise	0.028 (0.028)	0.019 (0.029)	0.024 (0.028)
Corporate size	-0.018 (0.038)	-0.033 (0.044)	-0.024 (0.044)
Beijing	0.139 (0.180)	0.075 (0.182)	0.095 (0.179)
Tianjin	-0.034 (0.159)	-0.002 (0.167)	-0.009 (0.164)
Hebei	0.003 (0.199)	0.032 (0.201)	-0.016 (0.199)
Shandong	0.099 (0.175)	0.087 (0.178)	0.111 (0.176)
**Main effects**			
Social culture		0.127 (0.090)	0.124 (0.089)
Experience of entrepreneurial failure		-0.143 (0.136)	-0.138 (0.133)
Business performance		0.005 (0.006)	0.003 (0.006)
**Interactive effects**			
Social culture ^∗^ experience of entrepreneurial failure			0.346^†^ (0.194)
Social culture ^∗^ business performance			0.017* (0.008)
R^2^	0.027	0.050	0.097
ΔR^2^	0.027	0.023	0.047*
F	0.438	0.637	1.110
VIFmax	2.145	2.222	2.269


*^†^*p* < 0.1, ^∗^*p* < 0.05; numbers in brackets refer to standard error.*

The multicollinearity tests on all equations showed that the maximum VIF of all variables is 2.269, which is far below the critical value of 5. All of the tolerances were above 0.4, which exceeds the critical value of 0.1. Therefore, no significant collinearity was shown between variables.

**FIGURE 1:**
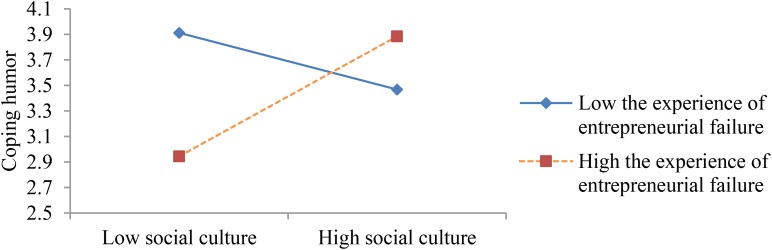
The interaction between social culture and entrepreneurial failure experience on coping humor.

**FIGURE 2:**
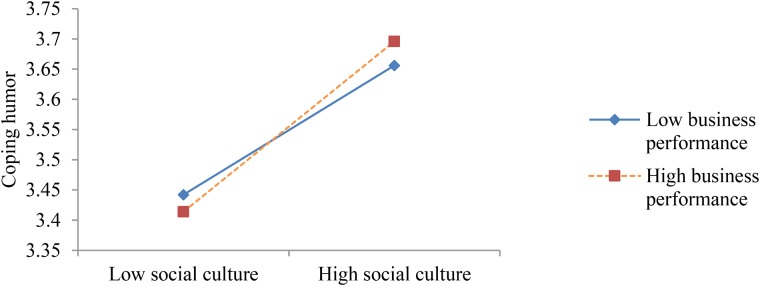
The interaction between social culture and business performance on coping humor.

The results of Model 3 show marginally significant interaction terms between social culture and the experience of entrepreneurial failure (*b* = 0.346, *p* = 0.076, <0.1) and significant interaction terms between social culture and business performance (*b* = 0.017, *p* = 0.039, <0.05). Therefore, H1 is marginally supported, and H2 is proven. When the interaction item was placed in Model 3, the adjusted R-square increased from 0.050 to 0.097 – that is, ΔR^2^ = 0.047 (*p* = 0.020, <0.05). The authors plotted the interactional pattern, as shown in **Figures 1**, **2**. **Figure 1** shows four points regarding the combination of social culture and the experience of entrepreneurial failure. Consistent with the authors’ hypotheses, people with “high social culture + high failure” report higher scores in coping humor than people with “high social culture + low failure” and people with “low social culture + high failure.” These scores indicate that the interaction between social culture and the experience of entrepreneurial failure had a slightly positive effect on entrepreneurs’ coping humor. People with “high culture + low failure” report lower scores in coping humor because, in that situation, although the perceived stress is lower, the sense of control over stress is lower too. Thus, entrepreneurs tend to deal with stress by using cautious or passive strategies rather than proactive ones (Folkman et al., 1979). People with “low culture + high failure” also report lower scores in coping humor because, in such circumstances, although the sense of control over stress is higher, the perceived stress is higher too. Thus, entrepreneurs may be inclined to use more careful and rational strategies than coping humor to explore solutions and solve problems as soon as possible, thereby showing their professional abilities and winning social support and recognition. Surprisingly, our results show that the scores for “low social culture + low failure” are similar to the scores for “high social culture + high failure,” which indicate that when the perceived stress is higher and the sense of control over stress is lower, entrepreneurs likely cope with stress by using coping humor. That strategy may be applied because the public does not support entrepreneurship, and failing to cope with stress through their own efforts, the entrepreneurs may turn to self-disparaging remarks to admit that they are incompetent.

**Figure 2** shows that the interaction between social culture and business performance had a positive effect on the coping humor of entrepreneurs. The results indicate that if the social group places a higher value on entrepreneurship and the enterprise has had better business performance, the entrepreneur will be more likely to use coping humor.

## Discussion

Based on coping theory, this paper explores the influences of interactions between social culture (affecting perceived stress) and entrepreneurial experience, including the experience of entrepreneurial failure and current business performance (affecting sense of control over stress) on the coping humor of entrepreneurs. The results show that if the social group highly appreciates entrepreneurship and the entrepreneur has experienced failure, the entrepreneur will be more likely to adopt coping humor; if the social group highly appreciates entrepreneurship and the enterprise has a better business performance, the entrepreneur will also be more likely to adopt coping humor.

It should be noted that the results show non-significant main effects of variables. Specifically, the direct influence of social culture on coping humor is not statistically significant. As mentioned above, social culture influences entrepreneurs’ perceived stress. If the social culture is more favorable to entrepreneurship, the perceived stress will be moderate (vs. high). In such situations, entrepreneurs may not find coping humor to be a necessary choice and may tend to deal with stress with a range of dynamic and diverse coping efforts such as making an action plan, self-adaptation, and drawing strength from adversity (Anderson, 1975). The direct influence of the experience of entrepreneurial failure on coping humor is statistically non-significant. As mentioned above, the experience of entrepreneurial failure influences entrepreneurs’ sense of control over stress. If entrepreneurs have experienced entrepreneurial failure, their sense of control over stress is higher than those who had not. Coping humor may not necessarily be a choice, and they tend to deal with stress by using proactive strategies such as seeking help and taking persistent actions to solve problems (McCrae, 1984).

The direct influence of current business performance on coping humor is statistically non-significant. Current business performance positively affects entrepreneurs’ sense of control over stress. As mentioned above, when current business performance is good, coping humor is also not necessarily a choice. In other words, although some researchers have stated that the degree of perceived stress and the sense of control over stress will affect the choice of coping strategies, respectively (Anderson, 1975; Folkman et al., 1979), our model argues that the interaction between the two factors affects entrepreneurs’ coping humor. This is because the social culture that affects the degree of perceived stress and the entrepreneurial experiences that reflect the sense of control over stress (including entrepreneurial failure and current business performance) directly affect entrepreneurs when using active strategies, but active strategies themselves are not immediately equivalent to coping humor. According to coping theory and our analysis, we believe that the combination of these two factors will urge entrepreneurs to take a more active and effective strategy, namely, coping humor rather than the usual active ones. Therefore, social culture and entrepreneurial experiences have interactive, rather than direct, effects on coping humor. In addition, studies on culture and cognition show that social culture and individual cognition have interactive effects on individual behaviors (Welter, 2011), and these are consistent with our studies.

There are many cases demonstrating the interaction between social culture and individual experiences. For instance, entrepreneurship support policies have gradually increased in China in recent years, and expansive positive publicity from the press has deepened the public’s understanding and created a positive reputation for entrepreneurship. This indicates that social culture has been more favorable to entrepreneurship in recent years. Entrepreneurs will face less pressure under such circumstances. In such a society, when confronting pressure, entrepreneurs who once failed as well as those who have achieved remarkable results will change their coping strategies from being serious and prudent to being humorous, for example through the use of self-mockery. The story of Luo Yonghao is a good case in point. He was the founder of a Chinese mobile phone brand and a serial entrepreneur who has experienced several failures, Luo ridicules himself in a humorous way when he is confronted with an entrepreneurial dilemma, which helps him gain public understanding and leaves a good impression on the public. Yu Minhong, the founder of the New Oriental Education & Technology Group, is a typical successful entrepreneur who always takes a humorous attitude in the face of setbacks. He wins public support and recognition by fostering a modest and tenacious public image.

### Theoretical Contributions

The paper has made the following theoretical contributions. First, by discussing the situations in which entrepreneurs use coping humor, the paper makes contributions to the theory of humor. Literature relating to humor has emphasized the key influence of humor on individual pressure as a coping mechanism. However, it is clear that humor is neither commonly used in the face of pressure (Yue, 2010; Wu and Chan, 2013) nor always effective (Overholser, 1992). Hence, no answers have been given by previous studies to the following two issues: first, which are the factors that influence an individual’s coping humor; and second, what are the situations in which coping humor can be more effective when dealing with pressure. While the latest literature on the usage of humor has mentioned that coping humor will be more effective for individuals under moderate pressure (Kim et al., 2016), further mechanism and empirical research is needed. The paper analyzes how social culture and individual experiences interact to affect the coping humor of entrepreneurs, thus making contributions to the development of the concepts and theories of humor and coping humor.

Second, the paper contributes to the literature relating to social culture that supports entrepreneurial activities. Social culture is a kind of institutional environment. Previous studies have taken the institutional environment faced by entrepreneurs as a variable at the macro level, believing that the institutional environment defines and limits entrepreneurial opportunities and affects the size and speed of new venture creation (Hwang and Powell, 2005). In particular, social culture refers to the values, beliefs, and attitudes shared by a social group, which determines the evaluation standard for organizational legitimacy and supports or constrains entrepreneurial activities and traces (Bruton et al., 2010). This study further expands the function of social culture, with its conclusion indicating that the interaction between social culture and entrepreneurs’ experience affects how they evaluate pressure as well as how they cope with it. This extends the function of social culture affecting entrepreneurs, a relatively macroscopic concept, on the emotion or cognition of entrepreneurs at the micro level, thus contributing to the literature relevant to social culture and the institutional environment.

Third, the paper adds to the literature on entrepreneurial failure. Although previous studies looked at the positive side of entrepreneurial failure, for example, providing learning opportunities and enhancing knowledge and skills (Shepherd, 2003; Cope, 2011; Parker, 2013), few have made in-depth analyses on how these advantages influence subsequent entrepreneurial cognition and actions of serial entrepreneurs. This research has discovered that the experience of entrepreneurial failure is related to the sense of control of serial entrepreneurs when they are faced with pressure, and that the experience of failure and the social culture that entrepreneurs perceive will interact on their strategy to cope with pressure. Therefore, this research contributes to literature relating to entrepreneurial failure by further exploring and proving the relationship between previous failure and subsequent entrepreneurial cognition and actions.

### Practical Significance

The study has considerable practical significance. First, pressure is often unavoidable for entrepreneurs. When facing relatively minor pressures and having a sense of control over them – for example, minor friction with subordinates, stakeholders, or the public – the entrepreneur should use coping humor as much as possible to defuse tension, ease others’ hostile or negative moods, improve their affinity, and cope with the event in a more efficient manner. Second, it is important for the public to have a better understanding of and support for entrepreneurship, especially understanding and cultivating tolerance toward entrepreneurial failure. This is not only beneficial to local entrepreneurship and economic development but, more importantly, also helps to reduce entrepreneurs’ perception of psychological pressure.

### Limitations and Prospects

This research has several limitations. First, the samples were only drawn from China’s Bohai Rim area. As social cultures in countries and regions differ, entrepreneurs will face various levels of psychological pressure from corresponding social groups, which limits horizontal comparisons between research results. In the future, there should be various sources of samples, and data should be collected from entrepreneurs from varying cultural backgrounds, if possible. Second, due to the lack of sufficient quantitative information, the data in this paper were mainly obtained from surveys. Future research should combine qualitative study with quantitative study to delve into complex issues relating to the coping humor of entrepreneurs. Third, the paper only makes a preliminary study on the interactive mechanism between factors affecting how entrepreneurs use coping humor under pressure, and the research framework adopted in this paper can be further improved, for instance, by introducing more variables at the individual level to describe how entrepreneurs cope with pressure. Fourth, our results show that entrepreneurs are more likely to adopt coping humor when they perceive social culture unsupportive to entrepreneurship and have not experienced entrepreneurial failure. That finding is beyond our theoretical expectation and also a deficiency of this study. We will further explore the mechanism of entrepreneurs’ coping humor in this situation.

## Author Contributions

SL made the theoretical design of this article and wrote some sections of the article. JL made the data analysis and wrote some sections of the article. RH made some analysis of the measure and wrote some sections of the article.

## Conflict of Interest Statement

The authors declare that the research was conducted in the absence of any commercial or financial relationships that could be construed as a potential conflict of interest.

## References

[B1] Akhtar R. Ahmetoglu G. Chamorro-Premuzic T. (2013). Greed is good? Assessing the relationship between entrepreneurship and subclinical psychopathy. *Pers. Individ. Dif.* 54 420–425. 10.1016/j.paid.2012.10.013

[B2] Anderson C. R. (1975). Stress, performance, and coping: a test of the inverted-u theme. *Acad. Manag. Proc.* 1975 152–154. 10.5465/ambpp.1975.4981065

[B3] Anderson C. R. (1977). Locus of control, coping behaviors, and performance in a stress setting: a longitudinal study. *J. Appl. Psychol.* 62 446–451. 10.1037/0021-9010.62.4.446 885833

[B4] Avloniti A. Iatridou A. Kaloupsis I. Vozikis G. S. (2014). Sibling rivalry: implications for the family business succession process. *Int. Entrep. Manag. J.* 10 661–678. 10.1007/s11365-013-0271-6

[B5] Bandura A. (1997). *Self-efficacy: The Exercise of Control*. New York, NY: Freeman.

[B6] Baum J. R. Locke E. A. Kirkpatrick S. A. (1998). A longitudinal study of the relation of vision and vision communication to venture growth in entrepreneurial firms. *J. Appl. Psychol.* 83 43–54. 10.1037/0021-9010.83.1.43

[B7] Benight C. C. Bandura A. (2003). Social cognitive theory of traumatic recovery: the role of perceived self-efficacy. *Behav. Res. Ther.* 42 1129–1148. 10.1016/j.brat.2003.08.008 15350854

[B8] Bhidé A. (2000). *The Origin and Evolution of New Businesses.* Oxford: Oxford University Press.

[B9] Bruton G. D. Ahlstrom D. Li H. L. (2010). Institutional theory and entrepreneurship: where are we now and where do we need to move in the future? *Entrep. Theory Pract.* 34 421–440. 10.1111/j.1540-6520.2010.00390.x

[B10] Busenitz L. W. Gómez C. Spencer J. W. (2000). Country institutional profiles: unlocking entrepreneurial phenomena. *Acad. Manag. J.* 43 994–1003.

[B11] Carayannopoulos S. (2009). How technology-based new firms leverage newness and smallness to commercialize disruptive technologies. *Entrep. Theory Pract.* 33 419–438. 10.1111/j.1540-6520.2009.00297.x

[B12] Cardon M. S. Stevens C. E. Potter D. R. (2011). Misfortunes or mistakes? : cultural sensemaking of entrepreneurial failure. *J. Bus. Venturing* 26 79–92. 10.1016/j.jbusvent.2009.06.004

[B13] Celso B. G. (2003). Humor coping, health status, and life satisfaction among older adults residing in assisted living facilities. *Aging Ment. Health* 7 438–445. 10.1080/13607860310001594691 14578005

[B14] Chapple A. Ziebland S. (2004). The role of humor for men with testicular cancer. *Qual. Health Res.* 14 1123–1139. 10.1177/1049732304267455 15359047

[B15] Connor-Smith J. K. Flachsbart C. (2007). Relations between personality and coping: a meta-analysis. *J. Pers. Soc. Psychol.* 93 1080–1107. 10.1037/0022-3514.93.6.1080 18072856

[B16] Cope J. (2011). Entrepreneurial learning from failure: an interpretative phenomenological analysis. *J. Bus. Venturing* 26 604–623. 10.1016/j.jbusvent.2010.06.002

[B17] Demjén Z. (2016). Laughing at cancer: humour, empowerment, solidarity and coping online. *J. Pragmat.* 101 18–30. 10.1016/j.pragma.2016.05.010

[B18] Ensley M. D. Pearson A. W. Amason A. C. (2002). Understanding the dynamics of new venture top management teams: cohesion, conflict, and new venture performance. *J. Bus. Venturing* 17 365–386. 10.1016/S0883-9026(00)00065-3

[B19] Fang H. C. Randolph R. V. D. G. Memili E. Chrisman J. J. (2016). Does size matter? the moderating effects of firm size on the employment of nonfamily managers in privately held family SMEs. *Entrep. Theory Pract.* 40 1017–1039. 10.1111/etap.12156

[B20] Folkman S. Schaefer C. Lazarus R. S. (1979). “Cognitive processes as mediators of stress and coping,” in *Human Stress and Cognition*, eds Hamilton V. Warburton D. M. (New York, NY: John Wiley & Sons), 265–298.

[B21] Gao J. Jiang Y. F. Li X. B. Cheng Y. (2005). *Global Entrepreneurship Monitor China Report.* Beijing: Tsinghua University Press.

[B22] Geisler F. C. M. Wiedig-Allison M. Weber H. (2009). What coping tells about personality. *Eur. J. Pers.* 23 289–306. 10.1002/per.709

[B23] Gilbert B. A. McDougall P. P. Audretsch D. B. (2006). New venture growth: a review and extension. *J. Manag.* 32 926–950. 10.1177/0149206306293860 12196045

[B24] Goetz T. Haag L. Lipnevich A. A. Keller M. M. Frenzel A. C. Collier A. P. M. (2014). Between-domain relations of students’ academic emotions and their judgments of school domain similarity. *Front. Psychol.* 5:1153. 10.3389/fpsyg.2014.01153 25374547 PMC4204457

[B25] Goldberg-Looney L. D. Sánchez-Sansegundo M. Ferrer-Cascales R. Albaladejo-Blazquez N. Perrin P. B. (2016). Adolescent alcohol use in Spain: connections with friends, school, and other delinquent behaviors. *Front. Psychol.* 7:269. 10.3389/fpsyg.2016.00269 26973567 PMC4776124

[B26] Gupta V. K. Turban D. B. Wasti S. A. Sikdar A. (2005). Entrepreneurship and stereotypes: are entrepreneurs from mars or from venus? *Acad. Manag. Annu. Meet. Proc.* 2005 C1–C6. 10.5465/ambpp.2005.18778633

[B27] Gupta V. K. Turban D. B. Wasti S. A. Sikdar A. (2009). The role of gender stereotypes in perceptions of entrepreneurs and intentions to become an entrepreneur. *Entrep. Theory Pract.* 33 397–417. 10.1111/j.1540-6520.2009.00296.x

[B28] Hwang H. Powell W. W. (2005). “Institutions and entrepreneurship,” in *Handbook of Entrepreneurship Research: Disciplinary Perspectives*, eds Alvarez S. A. Agarwal R. Sorenson O. (New York, NY: Springer), 201–232. 10.1007/0-387-23622-8_10

[B29] Kim S. Zhang X. A. Zhang B. W. (2016). Self-mocking crisis strategy on social media: focusing on alibaba chairman jack ma in china. *Public Relat. Rev.* 42 903–912. 10.1016/j.pubrev.2016.10.004

[B30] Kolympiris C. Kalaitzandonakes N. Miller D. (2015). Location choice of academic entrepreneurs: evidence from the US biotechnology industry. *J. Bus. Venturing* 30 227–254. 10.1016/j.jbusvent.2014.02.002

[B31] Kuiper N. A. Mckenzie S. D. Belanger K. A. (1995). Cognitive appraisals and individual differences in sense of humor: motivational and affective implications. *Pers. Individ. Dif.* 19 359–372. 10.1016/0191-8869(95)00072-E

[B32] Lazarus R. S. Folkman S. (1984). *Stress, Appraisal, and Coping*. New York, NY: Springer.

[B33] Lazarus R. S. Launier R. (1978). “Stress-related transactions between person and environment,” in *Perspectives in Interactional Psychology*, eds Pervin L. A. Lewis M. (London: Plenum Press).

[B34] Lefcourt H. M. (2001). *Humor: The Psychology of Living Buoyantly*. New York, NY: Kluwer Academic 10.1007/978-1-4615-4287-2

[B35] Lefcourt H. M. Davidson K. Prkachin K. M. Mills D. E. (1997). Humor as a stress moderator in the prediction of blood pressure obtained during five stressful tasks. *J. Res. Pers.* 31 523–542. 10.1006/jrpe.1997.2191

[B36] Lefcourt H. M. Martin R. A. (1986). *Humor and Life Stress: Antidote to Adversity*. New York, NY: Springer-Verlag 10.1007/978-1-4612-4900-9

[B37] Liu J. (2014). *Ma Yun Zi Chao Zhang Xiang: Wo Hen Pu Tong, Zhi Shi Zhang De Xiang Wai Xing Ren [Jack Ma self-mockery: I am ordinary. I just look like a ET]*. Available at: http://finance.ifeng.com/a/20141215/13356117_0.shtml

[B38] Liu Y. Almor T. (2016). How culture influences the way entrepreneurs deal with uncertainty in inter-organizational relationships: the case of returnee versus local entrepreneurs in china. *Int. Bus. Rev.* 25 4–14. 10.1016/j.ibusrev.2014.11.002

[B39] Manzano-García G. Ayala Calvo J. C. (2013). Psychometric properties of connor-davidson resilience scale in a Spanish sample of entrepreneurs. *Psicothema* 25 245–251. 10.7334/psicothema2012.183 23628541

[B40] Markman G. D. Balkin D. B. Baron R. A. (2002). Inventors and new venture formation: the effects of general self-efficacy and regretful thinking. *Entrep. Theory Pract.* 27 149–165. 10.1111/1540-8520.00004

[B41] Martin R. A. (1996). The situational humor response questionnaire (SHRQ) and coping humor scale (CHS): a decade of research findings. *Humor Int. J. Humor Res.* 9 251–272. 10.1515/humr.1996.9.3-4.251

[B42] Martin R. A. (2001). Humor, laughter, and Physical Health: methodological issues and research findings. *Psychol. Bull.* 127 504–519. 10.1037/0033-2909.127.4.504 11439709

[B43] Martin R. A. (2007). *The Psychology of Humor: An Integrative Approach*. Burlington, MA: Elsevier Academic Press.

[B44] Martin R. A. (2016). Humor and mental health. *Encycl. Ment. Health* 2 350–353. 10.1016/B978-0-12-397045-9.00044-6

[B45] Martin R. A. Kuiper N. A. Olinger L. J. Dance K. A. (1993). Humor, coping with stress, self-concept, and psychological well-being. *Humor Int. J. Humor Res.* 6 89–104. 10.1515/humr.1993.6.1.89

[B46] Martin R. A. Lefcourt H. M. (1983). Sense of humor as a moderator of the relation between stressors and moods. *J. Pers. Soc. Psychol.* 45 1313–1324. 10.1037/0022-3514.45.6.1313

[B47] Marziali E. McDonald L. Donahue P. (2008). The role of coping humor in the physical and mental health of older adults. *Aging Ment. Health* 12 713–718. 10.1080/13607860802154374 19023722

[B48] McCrae R. R. (1984). Situational determinants of coping responses: loss, threat, and challenge. *J. Pers. Soc. Psychol.* 46 919–928. 10.1037/0022-3514.46.4.919 6737200

[B49] McKelvie A. Haynie J. M. Gustavsson V. (2011). Unpacking the uncertainty construct: implications for entrepreneurial action. *J. Bus. Venturing* 26 273–292. 10.1016/j.jbusvent.2009.10.004

[B50] Merz E. L. Malcarne V. L. Hansdottir I. Furst D. E. Clements P. J. Weisman M. H. (2009). A longitudinal analysis of humor coping and quality of life in systemic sclerosis. *Psychol. Health Med.* 14 553–566. 10.1080/13548500903111798 19844834

[B51] Morse L. A. Xiong L. Ramirezzohfeld V. Anne S. Barish B. Lindquist L. A. (2018). Humor doesn’t retire: improvisation as a health-promoting intervention for older adults. *Arch. Gerontol. Geriatr.* 75 1–5. 10.1016/j.archger.2017.10.013 29156247

[B52] Murphy G. B. Trailer J. W. Hill R. C. (1996). Measuring performance in entrepreneurship research. *J. Bus. Res.* 36 15–23. 10.1016/j.ijnurstu.2007.11.004 18243207

[B53] Nett U. E. Goetz T. Hall N. C. (2011). Coping with boredom in school: an experience sampling perspective. *Contemp. Educ. Psychol.* 36 49–59. 10.1016/j.cedpsych.2010.10.003

[B54] Overholser J. C. (1992). Sense of humor when coping with life stress. *Pers. Individ. Dif.* 13 799–804. 10.1016/0191-8869(92)90053-R

[B55] Parker S. C. (2013). Do serial entrepreneurs run successively better-performing businesses? *J. Bus. Venturing* 28 652–666. 10.1016/j.jbusvent.2012.08.001

[B56] Pe’er A. Vertinsky I. Keil T. (2016). Growth and survival: the moderating effects of local agglomeration and local market structure. *Strateg. Manag. J.* 37 541–564. 10.1002/smj.2331

[B57] Robins R. W. Hendin H. M. Trzesniewski K. H. (2001). Measuring global self-esteem: construct validation of a single-item measure and the Rosenberg self-esteem scale. *Pers. Soc. Psychol. Bull.* 27 151–161. 10.1177/0146167201272002

[B58] Schendel D. (2007). Risk and uncertainty. *Strateg. Entrep. J.* 1 53–55. 10.1002/sej.17

[B59] Schonfeld I. S. Mazzola J. J. (2015). A qualitative study of stress in individuals self-employed in solo businesses. *J. Occup. Health Psychol.* 20 501–513. 10.1037/a0038804 25705913

[B60] Scott W. R. (2007). *Institutions and Organizations: Ideas and Interests*. Thousand Oaks, CA: Sage Publications.

[B61] Shane S. Cable D. (2002). Network ties, reputation, and the financing of new ventures. *Manag. Sci.* 48 364–381. 10.1287/mnsc.48.3.364.7731

[B62] Shepherd D. A. (2003). Learning from business failure: propositions of grief recovery for the self-employed. *Acad. Manag. Rev.* 28 318–328. 10.5465/amr.2003.9416377

[B63] Singh K. Mitchell W. (2005). Growth dynamics: the bidirectional relationship between interfirm collaboration and business sales in entrant and incumbent alliances. *Strateg. Manag. J.* 26 497–521. 10.1002/smj.462

[B64] Sliter M. Kale A. Yuan Z. (2014). Is humor the best medicine? The buffering effect of coping humor on traumatic stressors in firefighters. *J. Organ. Behav.* 35 257–272. 10.1002/job.1868

[B65] Toman W. (1992). *Family Constellation: Its Effects on Personality and Social Behavior*. New York, NY: Springer Pub. Co.

[B66] Ucbasaran D. Shepherd D. A. Lockett A. Lyon J. (2013). Life after business failure: the process and consequences of business failure for entrepreneurs. *J. Manag.* 39 163–202. 10.1177/0149206312457823

[B67] Ucbasaran D. Westhead P. Wright M. (2006). Habitual entrepreneurs experiencing failure: overconfidence and the motivation to try again. *Adv. Entrep. Firm Emerg. Growth* 9 9–28. 10.1016/S1074-7540(06)09002-7

[B68] Ucbasaran D. Westhead P. Wright M. Flores M. (2010). The nature of entrepreneurial experience, business failure and comparative optimism. *J. Bus. Venturing* 25 541–555. 10.1016/j.jbusvent.2009.04.001

[B69] Wanous J. P. Reichers A. E. Hudy M. J. (1997). Overall job satisfaction: how good are single-item measures? *J. Appl. Psychol.* 82 247–252. 10.1037/0021-9010.82.2.247 9109282

[B70] Welter F. (2011). Contextualizing entrepreneurship—conceptual challenges and ways forward. *Entrep. Theory Pract.* 35 165–184. 10.1111/j.1540-6520.2010.00427.x

[B71] Wiklund J. Davidsson P. Delmar F. (2003). What do they think and feel about growth? An expectancy-value approach to small business managers’ attitudes toward growth. *Entrep. Theory Pract.* 27 247–270. 10.1111/1540-8520.t01-1-00003

[B72] Wu J. Chan R. M. (2013). Chinese teachers’ use of humour in coping with stress. *Int. J. Psychol.* 48 1050–1056. 10.1080/00207594.2012.734623 23126310

[B73] Xie J. L. Schaubroeck J. Lam S. S. K. (2008). Theories of job stress and the role of traditional values: a longitudinal study in China. *J. Appl. Psychol.* 93 831–848. 10.1037/0021-9010.93.4.831 18642987

[B74] Yamakawa Y. Peng M. W. Deeds D. L. (2015). Rising from the ashes: cognitive determinants of venture growth after entrepreneurial failure. *Entrep. Theory Pract.* 39 209–236. 10.1111/etap.12047

[B75] Yue X. Jiang F. Lu S. Hiranandani N. (2016). To be or not to be humorous? cross cultural perspectives on humor. *Front. Psychol.* 7:1495. 10.3389/fpsyg.2016.01495 27757091 PMC5048456

[B76] Yue X. D. (2010). Exploration of Chinese humor: historical review, empirical findings, and critical reflections. *Humor Int. J. Humor Res.* 23 403–420.

